# Metabolism of Amino Acids in Cancer

**DOI:** 10.3389/fcell.2020.603837

**Published:** 2021-01-12

**Authors:** Zhen Wei, Xiaoyi Liu, Chunming Cheng, Wei Yu, Ping Yi

**Affiliations:** ^1^Hubei Province Key Laboratory of Occupational Hazard Identification and Control, School of Medicine, Brain Science and Advanced Technology Institute, Wuhan University of Science and Technology, Wuhan, China; ^2^Department of Obstetrics and Gynecology, The Third Affiliated Hospital of Chongqing Medical University, Chongqing, China; ^3^Department of Radiation Oncology, James Comprehensive Cancer Center and College of Medicine at The Ohio State University, Columbus, OH, United States; ^4^State Key Laboratory of Genetic Engineering, School of Life Sciences, Zhongshan Hospital, Fudan University, Shanghai, China

**Keywords:** amino acids (AAs), metabolism, cancer, mTORC (mammalian target of rapamycin kinase complex), epigenetic, tumor immunity, ferroptosis, tumor growth

## Abstract

Metabolic reprogramming has been widely recognized as a hallmark of malignancy. The uptake and metabolism of amino acids are aberrantly upregulated in many cancers that display addiction to particular amino acids. Amino acids facilitate the survival and proliferation of cancer cells under genotoxic, oxidative, and nutritional stress. Thus, targeting amino acid metabolism is becoming a potential therapeutic strategy for cancer patients. In this review, we will systematically summarize the recent progress of amino acid metabolism in malignancy and discuss their interconnection with mammalian target of rapamycin complex 1 (mTORC1) signaling, epigenetic modification, tumor growth and immunity, and ferroptosis. Finally, we will highlight the potential therapeutic applications.

## Introduction

Metabolic reprogramming is one of the hallmarks of cancer (Pavlova and Thompson, 2016; Revathidevi and Munirajan, 2019; Faubert et al., 2020; Hoxhaj and Manning, 2020; Leone and Powell, 2020). Increased approaches to killing cancer cells have been investigated by obstructing different metabolic pathways in tumors (Altman et al., 2016; Luengo et al., 2017; Vander Heiden and Deberardinis, 2017; Leone et al., 2019). Among them, the metabolic networks of all amino acids are complex and highly interconnected with other pathways (Li and Zhang, 2016). Amino acid metabolism has extremely extensive effects in cancer cells, including, but not limited to, (1) establishing amino acid pools as building blocks, especially the production of non-essential amino acids for protein biosynthesis, conversion to glucose, lipids, and precursors of nitrogen-containing metabolites, such as purines and pyrimidines for nucleic acid synthesis; as nutrient signals, to activate important pathways [mammalian target of rapamycin complex (mTORC) and autophagy]; or as neurotransmitters, such as glycine and D-serine; (2) epigenetic modification, such as methyl donor S-adenosyl methionine (SAM) from the methionine cycle; (3) bioenergy supply through producing α-ketoacid, which is ultimately oxidized by the tricarboxylic acid (TCA) cycle and oxidative phosphorylation for ATP production; (4) detoxification of ammonia by conversion to non-toxic urea; and (5) maintaining intracellular redox status (e.g., synthesis of the major non-enzymatic cellular antioxidant glutathione, from glutamate, cysteine, and glycine). Hence, abnormal amino acid metabolism has diverse and important roles in various cancers, and the potential impact of metabolic control and regulation in the tumor microenvironment is becoming increasingly important. In this review, we will systematically summarize the recent progress in amino acid metabolism in the context of cancer and the tumor microenvironment, describe their interconnection or indispensable role in cancer growth, epigenetic modification, cancer immunity, and ferroptosis, and discuss the potential therapeutic applications for targeting amino acid metabolism.

## Amino Acid Metabolism in Cancer

### Glutamine Metabolism

Glutamine is a non-essential amino acid, but many tumor cells depend on extracellular glutamine for survival. As such, glutamine is recognized as a conditionally essential amino acid. Glutamine is used as a major nitrogen source and carbon source to synthesize amino acids, lipids, and nucleic acids. Glutamine is imported into cancer cells via multiple transporters, including the Na^+^-dependent transporters, system ASC (alanine/serine/cysteine-preferring) that function as obligatory exchangers, and the Na^+^-coupled neutral amino acid transporters (SNATs), which belong to the SLC38 superfamily (Cha et al., 2018; Kandasamy et al., 2018). Among them, ASCT2 (*SLC1A5*), SNAT1 (*SLC38A1*) (system A), SNAT2 (*SLC38A2*) (system A), and SNAT5 (*SLC38A5*) (system N) have been found to be highly expressed in tumors (Bhutia et al., 2015). Pharmacological or genetic inhibition of ASCT2 (*SLC1A5*) has been shown to reduce the growth of gastric cancer (Lu J. et al., 2017), prostate cancer (Wang Q. et al., 2015), and triple-negative breast cancer (Van Geldermalsen et al., 2016). However, as triggering the compensatory responses, blocking a single glutamine transporter is not enough to prevent tumor growth. As proof, ASCT2 (*SLC1A5*) knockdown in osteosarcoma and cervical cancer cells can induce upregulation of SNAT1 (*SLC38A1*) (Broer et al., 2016).

**Figure 1:**
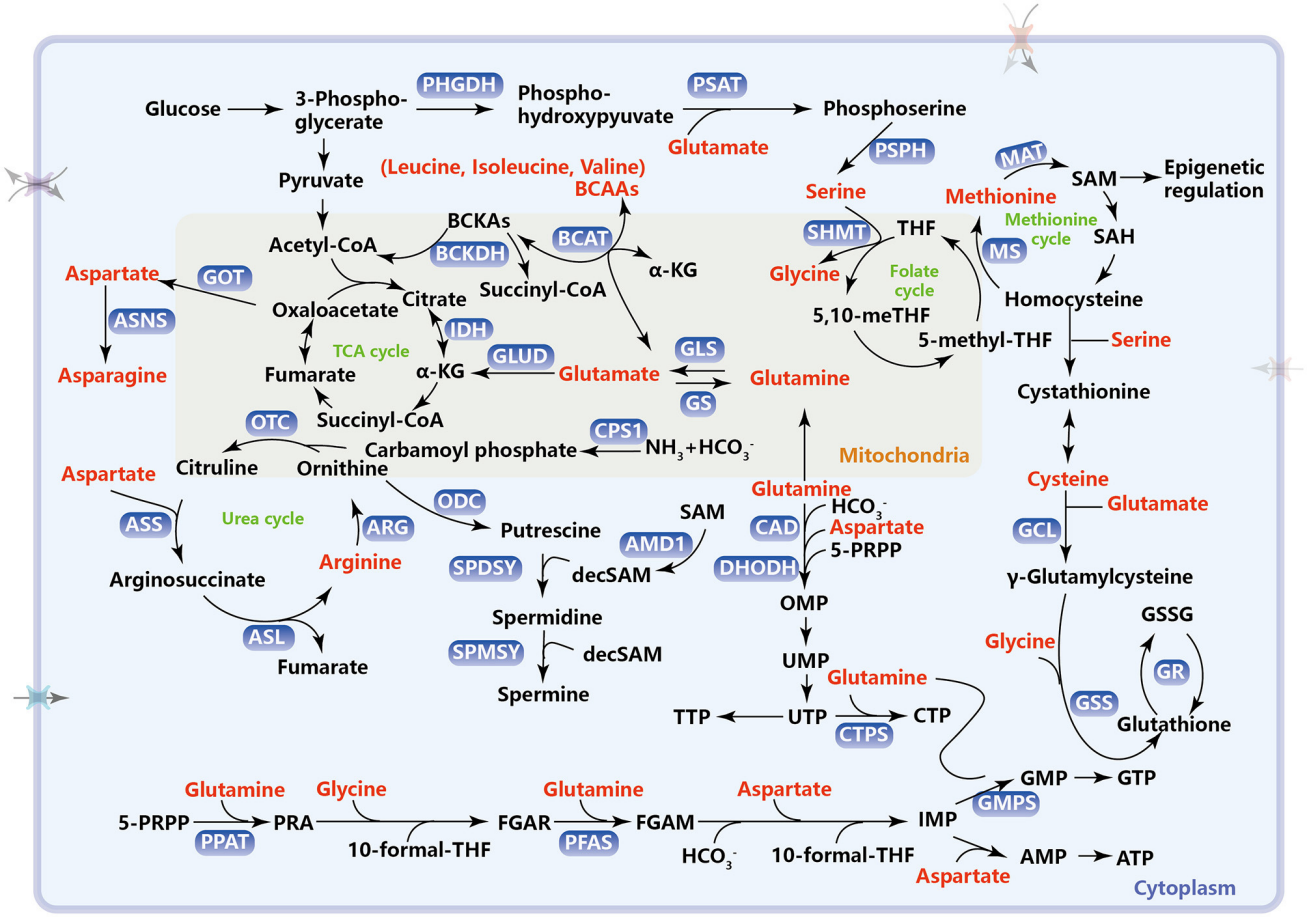
Metabolic pathways of amino acids in cancer. This schematic diagram briefly summarizes the amino acid metabolism which includes one-carbon metabolism, TCA cycle, urea cycle, and intracellular antioxidant, reduced glutathione synthesis. PHGDH, phosphoglycerate dehydrogenase; PSAT, phosphoserine aminotransferase; PSPH, phosphoserine phosphatase; SHMT, serine hydroxymethyltransferase; THF, tetrahydrofolate; MS, methionine synthetase; MAT, methionine adenosine transferase; SAM, S-adenosylmethionine; SAH, S-adenosylhomocysteine; BCAAs, branched-chain amino acids; BCAT, branched-chain amino transferase; BCKAs, branched-chain α-ketoacids; BCKDH, BCKA dehydrogenase enzyme complex; GOT, aspartate transaminase; ASNS, asparagine synthetase; GLS, glutaminase; GS, glutamine synthetase; GLUD, glutamate dehydrogenase; IDH, isocitrate dehydrogenase; TCA, tricarboxylic acid; CPS1, carbamoyl phosphate synthetase 1; ARG, arginase; OTC, ornithine transcarboxylase; ASS, argininosuccinate synthase; ASL, argininosuccinate lyase; ODC, ornithine decarboxylase; SPDSY, spermidine synthase; SPMSY, spermine synthase; decSAM, decarboxylated S-adenosylmethionine; AMD1, adenosylmethionine decarboxylase 1; GCL, glutamylcysteine ligase; GSS, glutathione synthetase; GR, glutathione reductase; GSSG, glutathione disulfide; CAD, carbamoylphosphate synthetase, including carbamoyl phosphate synthetase 2, aspartate transcarbamylase, and dihydrooratase; DHODH, dihydroorotate dehydrogenase; 5-PRPP, 5-phosphoribosyl pyrophosphate; OMP, orotidine 5′-monophosphate; UMP, uridine monophosphate; UTP, uridine triphosphate; CTP, cytidine triphosphate CTPS, cytidine triphosphate synthase; PPAT, phosphoribosyl pyrophosphate amidotransferase; PRA, phosphoribosyl-β-amine; FGAR, formylglycinamide ribonucleotide; PFAS, phosphoribosylformylglycinamidine synthase; FGAM, formylglycinamidine ribonucleotide; IMP, inosine monophosphate; GMP, guanosine monophosphate; GMPS, guanosine monophosphate synthase; GTP, guanosine triphosphate; AMP, adenosine monophosphate; ATP, adenosine triphosphate.

Glutamine catabolism, namely glutaminolysis, begins with its conversion to glutamate, which is catalyzed by the glutaminase (GLS) (Hassanein et al., 2013; Mates et al., 2013). Glutamate can be further converted to α-ketoglutarate (α-KG) through oxidative deamination by glutamate dehydrogenase (GLUD1) or transamination by glutamate-linked transaminase as an anaplerotic substrate in the TCA cycle for energy production (Figure 1). Many tumor cells are highly dependent on glutamine to supplement and renew the TCA cycle (Yang et al., 2017). α-KG can also be exported to the cytosol to be carboxylated into citrate through isocitrate dehydrogenase 1 (IDH1) and further into acetyl-CoA for *de novo* fatty acid synthesis (Fendt et al., 2013). Particularly, tumor growth under hypoxia or mitochondrial dysfunction relies almost exclusively on this reductive glutamine metabolism for lipid biosynthesis (Metallo et al., 2011). Furthermore, glutamine-derived fumarate, malate, and citrate are significantly increased when glucose is deprived, demonstrating that glutamine drives the glucose-independent TCA cycle in a nutrient-poor tumor microenvironment (Le et al., 2012). Glutamine also functions as both a carbon and nitrogen donor for the synthesis of reduced glutathione (GSH) by providing glutamate and enabling cysteine uptake (Wu et al., 2004; Conrad and Sato, 2012) (the role of glutaminolysis in GSH synthesis is addressed in section Amino Acid Metabolism, ROS, and Ferroptosis). Recently, a research report has shown that glutamine deficiency changes mitochondrial morphology and GLS1, as the glutamine sensor, participates in triggering mitochondrial fusion in a non-enzymatic manner (Cai et al., 2018). As a nitrogen donor, glutamine also provides an amide (γ-nitrogen) group to enable the *de novo* synthesis of nucleotides, which functions as a rate-limiting factor in cancer cell proliferation (Cox et al., 2016; Metzler et al., 2016; Yang et al., 2017; Wang et al., 2019a). Glutamine, along with aspartate and bicarbonate, is used as a substrate for the synthesis of the pyrimidine ring. This process is catalyzed by carbamoylphosphate synthetase (CAD). Namely, carbamoyl phosphate synthetase 2 (CPS 2) can accept the amide group from glutamine to generate carbamoyl phosphate, a rate-limiting step in pyrimidine synthesis. Then, the pyrimidine ring and 5-pyriphosphoribosyl pyrophosphate (5-PRPP), generated from the pentose phosphate pathway (PPP), are catalyzed to form orotidine 5′-monophosphate (OMP) via dihydroorotate dehydrogenase (DHODH). OMP continues to be converted into orotidine monophosphate (UMP) and then uridine triphosphate (UTP), which is then converted into CTP by cytidine triphosphate synthetase (CTPS) via utilizing the amide group on glutamine (Figure 1). For purine synthesis, 5-PRPP is converted into phosphoribosyl-β-amine (PRA) with the addition of an amide group from glutamine by phosphoribosyl pyrophosphate amidotransferase (PPAT). Furthermore, the amide group can also be transferred to formylglycinamide ribonucleotide (FGAR), which in turn forms formylglycinamidine ribonucleotide (FGAM) for nucleotide synthesis by phosphoribosylformylglycinamidine synthase (PFAS). Glutamine, glycine, aspartate, 10-formyl THF, and CO_2_ are used as substrates for the synthesis of inosine monophosphate (IMP)—the intermediate product of AMP and GMP (Figure 1).

**Figure 2:**
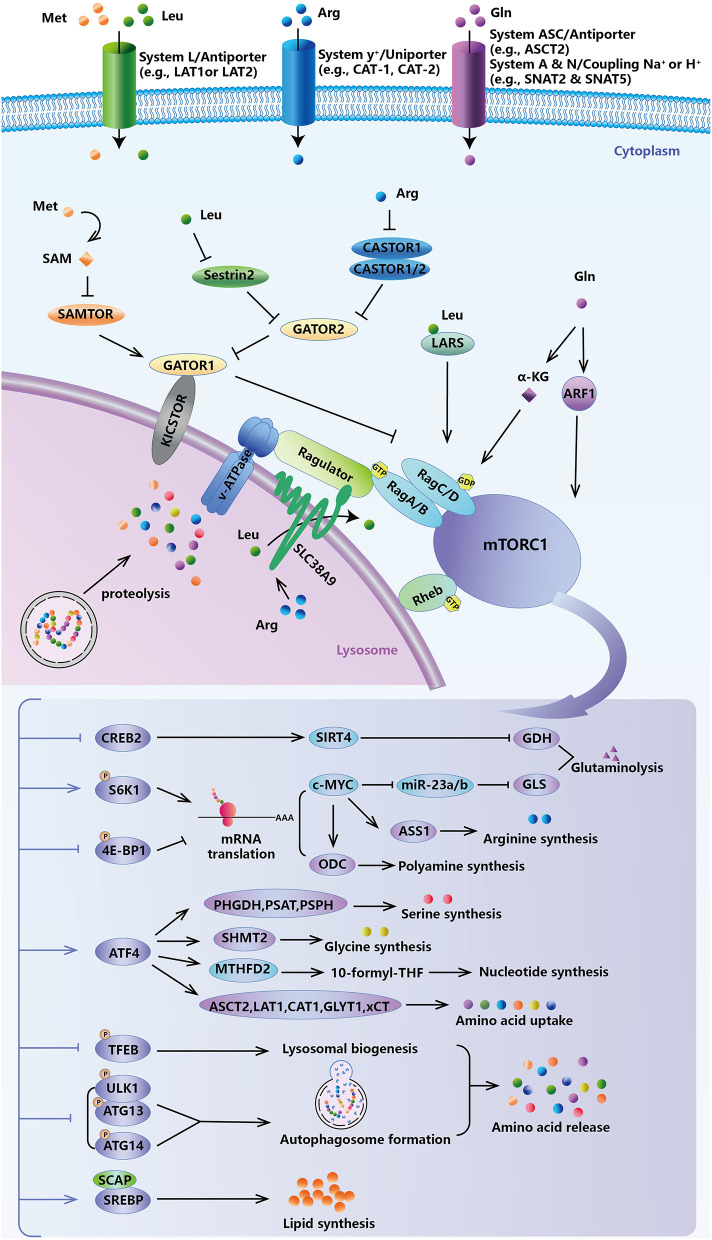
Feedback of amino acid-sensing mTORC1 signal. mTORC1 governs cancer cells to sense fluctuations in extracellular and intracellular amino acids, and to in turn modulate intracellular levels of amino acids, lipids, and nucleotides for survival and growth demand.

Several studies have shown that oncogenic and anti-oncogenic alterations in cancer cells may reprogram glutamine metabolism. Oncogenic Myc reorients mitochondrial metabolism, making it highly dependent on exogenous glutamine for cell survival (Wise et al., 2008). Accordingly, glutamine deprivation selectively induces apoptosis in MYC-amplified cancer cells (Qing et al., 2012). Because c-Myc mediated miR-23a and miR-23b repression, overexpression of c-MYC can upregulate GLS, and therefore promote glutaminolysis (Gao et al., 2009) (Figure 2). However, c-MYC can also increase demethylation of the glutamine synthetase (GS) promoter under glutamine limitation and induce high expression of GS (Bott et al., 2015). Notably, c-Myc was also reported to transcriptionally increase the expression of high-affinity glutamine transporters, including ASCT2 (*SLC1A5*) and SNAT5 (*SLC38A5*) (Wise et al., 2008; Perez-Escuredo et al., 2016; Zhao et al., 2019). In pancreatic cancer, the Kirsten rat sarcoma viral oncogene homolog (KRAS) gene reprograms glutamine metabolism by upregulating aspartate transaminase (GOT1) and repressing GLUD1 to increase the NADPH/NADP(+) ratio for stable redox capacity (Son et al., 2013). In KRAS-mutant cells, glutamine can enhance the oxygen consumption and ATP production to promote tumorigenesis (Weinberg et al., 2010). More importantly, glutamine metabolism is also regulated by tumor suppressors. The glutaminase encoded by GLS2 can be directly bound and transcriptionally induced by p53, reducing cellular sensitivity to ROS-associated apoptosis, possibly through glutathione-dependent antioxidant defense (Suzuki et al., 2010; Mates et al., 2013). Deficiency of liver kinase B1 (LKB1) may increase glucose and glutamine uptake and utilization in a HIF1α-dependent way (Faubert et al., 2014). Another tumor suppressor, retinoblastoma (Rb), can directly regulate the glutamine transporter ASCT2 (*SLC1A5*) to reduce glutamine uptake via the transcription factor E2F3 (Reynolds et al., 2014). These metabolic regulations meet the high glutamine demand of proliferating tumor cells, which supports tumor growth by facilitating both energy production and the biosynthesis of building materials.

### Serine and Glycine Metabolism

Like glucose, amino acids are also important for the synthesis of building blocks, including proteins, nucleic acids, and lipids, that are crucial to cancer cell proliferation (Murugan, 2019; Sivanand and Vander Heiden, 2020; Vettore et al., 2020). Serine, as a one-carbon source in nucleotide synthesis and DNA methylation, plays an important role in cancer progression. Many tumors depend on the availability of extracellular serine for rapid proliferation. Serine starvation inhibits the proliferation of colorectal cancer cells in *in vitro* (Labuschagne et al., 2014; Maddocks et al., 2016) and tumor growth in *in vivo* (Maddocks et al., 2013). Moreover, restriction of serine and glycine intake can inhibit tumor growth and extend the survival time of tumor-bearing mice (Maddocks et al., 2017). More interestingly, this effect is more pronounced in tumor cells with a p53 deficiency (Maddocks et al., 2013). Serine is a small, neutral amino acid and can be imported into the cell by Na^+^-dependent transporters like ASCT1 (*SLC1A4*), which is upregulated in both breast cancer (Pollari et al., 2011) and lung cancer (Riscal et al., 2016), system A transporters like SNAT1 (*SLC38A1*), and the ASC system (El-Hattab, 2016). However, whether the exogenous serine or glycine supports the proliferation of cancer cells is still highly debatable. Jain et al. (2012) identified glycine as a key metabolite for rapid cancer cell proliferation, but Labuschagne et al. (2014) theorized that nucleotide synthesis and cancer cell proliferation are supported by serine rather than glycine consumption. Thus, it is possible that different cancer types have specific nutrition addictions (Altman et al., 2016; Bernfeld and Foster, 2019; Maggi and Scotti, 2019; Vettore et al., 2020).

Besides importing from the extracellular environment, cells can synthesize serine from intracellular glucose through the *de novo* serine synthesis pathway (SSP), which is considered necessary for cancer cells (Locasale, 2013). In SSP, 3-phosphoglycerate (3-PG), the intermediate metabolite of glycolysis, is first converted to 3-phosphohydroxypyruvate (3-PH), which is catalyzed by 3-phosphoglycerate dehydrogenation (PHGDH). 3-PH can obtain an amino group from glutamate and form 3-phosphoserine (3-PS) through phosphoserine transaminase (PSAT) followed by dephosphorylation through serine phosphatase (PSPH) to finally produce serine (Figure 1). The three SSP enzymes (PHGDH, PSAT, and PSPH) are all highly expressed in various cancers to meet the large serine demand for survival (Locasale et al., 2011; Pollari et al., 2011; Possemato et al., 2011; Zhang et al., 2012). In KRAS-mutant cancer cells with or without LKB1 loss, increased expression of SSP genes and high serine synthesis allow the cells to tolerate serine starvation for survival (Kottakis et al., 2016; Maddocks et al., 2017). A few transcription factors are reported to promote activation of SSP genes. Activating transcription factor 4 (ATF4) can directly bind to the promoters of both PHGDH and PSAT1 and promote their expression under amino acid deprivation and hypoxia (Ye et al., 2012; Denicola et al., 2015). In addition, lysine demethylase 4C (KDM4C) can also increase the expression of SSP genes by directly demethylating the promoter of SSP genes or ATF4, a transcriptional substrate of NRF2 (Denicola et al., 2015; Zhao et al., 2016). However, when apoptosis occurs, p53 may inhibit PHGDH transcription and reduce unnecessary serine synthesis (Ou et al., 2015). Serine, the end-product of the SSP pathway, is an effective regulator of serine synthesis. Serine from SSP can directly bind to pyruvate kinase M2 (PKM2), the last rate-limiting enzyme of glycolysis, and promote the allosteric activation of PKM2 for the positive feedback of continuously activated glycolysis and serine synthesis (Chaneton et al., 2012; Ye et al., 2012). By contrast, serine itself is an allosteric inhibitor of PHGDH, and this allosteric inhibition can spontaneously disappear at low serine levels (Sugimoto and Pizer, 1968). In all, SSP genes and the serine synthesis pathway are activated under tumor context and can be regulated by intracellular serine levels.

Serine hydroxymethyltransferase (cytoplasmic, SHMT1; mitochondrial, SHMT2) catalyzes the transfer of the beta carbon of serine to tetrahydrofolate (THF) to form glycine and one-carbon units, 5,10-methylene-THF (Figure 1), which is critical for nucleotide synthesis. Glycine can also be cleaved by the mitochondrial glycine cleavage system to yield 5,10-methylene-THF, which enters the folate cycle (Tibbetts and Appling, 2010). Both glycine and 5,10-methylene-THF, the two reaction products catalyzed by SHMT, provide two carbon atoms for the purine ring through a series of one-carbon unit conversions (Labuschagne et al., 2014; Pacold et al., 2016; Ma et al., 2017). Although one-carbon metabolism occurs in both the cytoplasm and mitochondria, metabolic enzymes, including SHMT in mitochondria but not the cytosol, are upregulated and significantly associated with tumor aggressiveness and prognosis (Jain et al., 2012; Lee et al., 2014; Nilsson et al., 2014). Given that mitochondrial one-carbon metabolism is very important for cancer progression, the entry of serine into the mitochondria is a critical step in the generation of one-carbon units. The mitochondrial transporter(s) for serine was first identified as sideroflexin 1 (SFXN1) by David M. Sabatini and his colleagues in 2018 (Kory et al., 2018). As the key metabolic enzymes, SHMTs are the direct transcriptional targets of c-Myc (Nikiforov et al., 2002; Nilsson et al., 2012), and SHMT2 can be co-induced by c-Myc and HIF1α to maintain NADPH production and redox balance in neuroblastoma and breast cancer cells (Ye et al., 2014). SHMT2 not only was found to be highly expressed in glioblastoma (GBM) (Kim et al., 2015), hepatocellular carcinoma (HCC) (Woo et al., 2016), colorectal cancer (CRC) (Wei et al., 2018), and diffuse large B-cell lymphoma (DLBCL) (Ducker et al., 2017) but also specifically promoted the growth and proliferation of tumor cells when the glycine cleavage system was not functioning properly (Kim et al., 2015). Recently, the post-translation modifications of SHMT2 have been extensively explored. The deacetylation of SHMT2 at K95 by SIRT3 (Wei et al., 2018) or desuccinylation at K280 by SIRT5 (Yang et al., 2018) has been shown to enhance the enzymatic activity of this protein, and the deacetylation by SIRT3 also has been shown to stabilize SHMT2 through avoiding its autophagic degradation (Wei et al., 2018).

As drivers of cancer pathogenesis, the serine, glycine, one-carbon (SGOC) metabolic network is widely appreciated (Locasale, 2013). This metabolic network contributes to methylation of DNA/RNA and the *de novo* ATP synthesis in cancer cells (Maddocks et al., 2016). In addition, it directly modulates adaptive immunity by controlling effector T-cell expansion and activation (Ma et al., 2017). For proper mitochondrial translation of respiratory chain enzymes, the SGOC network also occupies an indispensable position by maintaining the formylation of initiator tRNAs (Minton et al., 2018; Morscher et al., 2018; Tani et al., 2018). In all, serine and glycine metabolism could yield a promising set of potential targets for cancer therapy.

### Branched-Chain Amino Acid (BCAA) Metabolism

The branched-chain amino acids (BCAAs)—leucine, isoleucine, and valine—are essential amino acids for mammals. BCAAs cannot be synthesized by human cells, but are obtained via dietary intake and scavenged protein recycling (Neinast M. D. et al., 2019). BCAAs account for about 63% of the hydrophobic amino acids in mammalian proteins (Neinast M. et al., 2019). LAT1 (*SLC7A5*) is the main BCAA transporter, which belongs to the Na^+^- and pH-independent L-type amino acid transporters (system L/antiporter) SLC7 family and is highly expressed in many cancers, including the most frequently diagnosed cancers, such as lung cancer, prostate cancer, and breast cancer (Kandasamy et al., 2018; Singh and Ecker, 2018; Hafliger and Charles, 2019). Notably, a recent study found that LAT1 was a pH-dependent but not pH-independent transporter, whose maximal transporter activity is at neutral pH but much lower at acidic pH (Cosco et al., 2020). LAT1 (*SLC7A5*) imports BCAAs and several essential amino acids (e.g., phenylalanine, leucine, isoleucine, tryptophan, histidine, and tyrosine) with high affinity in exchange for the efflux of intracellular histidine, tyrosine, and glutamine (Nicklin et al., 2009; Puris et al., 2020). To maintain amino acid nutrition for tumor growth, the expression of the LAT1 (*SLC7A5*) transporter is controlled by the pro-carcinogenic transcription factors c-Myc (Yue et al., 2017), HIF2α (Elorza et al., 2012), and NOTCH (Grzes et al., 2017), as well as the post-transcriptional regulator miR-126 (Miko et al., 2011). In prostate cancer, expression of androgen receptor–mediated LAT3 (*SLC43A1*) and leucine uptake can be inhibited by anti-androgen treatment, inducing the compensatory upregulation of LAT1 (*SLC7A5*) (Wang et al., 2011). LAT2 (*SLC7A8*), another important BCAA transporter, has been reported to be abnormally expressed in cancers (Wang and Holst, 2015). In pancreatic cancer, LAT2 (*SLC7A8*) was shown to decrease gemcitabine sensitivity by regulating glutamine-dependent mTOR activation to promote proliferation and inhibit apoptosis (Feng et al., 2018). Recently, several specific small molecule inhibitors of LAT1 (*SLC7A5*) and LAT2 (*SLC7A8*) have been discovered, which facilitate the clinical application of system L transporters (Table 1; Zaugg et al., 2020).

**Table 1:** Key enzymes/transporters and their drugs in cancer amino acid metabolism.

	**Target**	**Drug**	**Mechanism**	**Cancer type**	**Clinical phases**
Glutamine metabolism	GLS1	CB-839	Inhibiting GLS1 activity	Non-small cell lung cancer, myeloma (Momcilovic et al., 2017; Thompson et al., 2017)	Phase I and II
	GLS	BPTES	Inhibiting GLS activity	Hepatocellular carcinoma, B-cell lymphoma (Xiang et al., 2015), pancreatic cancer (Elgogary et al., 2016)	
	GLS1	968	Inhibiting GLS1 activity	Non-small cell lung cancer (Han et al., 2017)	
Serine and one-carbon metabolism	PHGDH	CBR-5884	Disrupting the oligomerization state of PHGDH	Breast cancer, melanoma (Mullarky et al., 2016)	
	PHGDH	NCT-503	Inhibiting PHGDH activity	Breast cancer (Pacold et al., 2016)	
	SHMT1/2	SHIN1	Inhibiting SHMT1/2 activity	Diffuse large B-cell lymphoma (Ducker et al., 2017)	
	MAT2A	FIDAS-5	Competing against SAM for MAT2A binding	Lung cancer (Wang Z. et al., 2019)	
	RNR	Gemcitabine	Disrupting DNA replication/synthesis	Pancreatic cancer (Kamisawa et al., 2016)	Approved
	TYMS	5-FU	Blocking thymidylate synthase	Colorectal cancer (Ser et al., 2016)	Approved
	DHFR, MTHFR	Methotrexate	Inhibiting DHFR and purine/pyrimidine base biosynthesis	Acute leukemia (Winter et al., 2018)	Approved
	DHFR, TYMS, MTHFR	Pemetrexed	A folate analog, inhibiting DHFR, TYMS, and MTHFR	Non-small cell lung carcinoma, pleural mesothelioma (Beddowes et al., 2017)	Approved
	DHFR	Pralatrexate	A folate analog, inhibiting DHFR	Peripheral T-cell lymphoma (Amengual et al., 2018)	Approved
	DHFR, TYMS	Raltitrexed	A folate analog, inhibiting DHFR, TYMS	Colorectal cancer (Newman and Maddocks, 2017)	Approved
Arginine metabolism	Arginine deprivation	ADI-PEG20	The recombinant human pegylated arginine deiminase	Hepatocellular carcinoma (Abou-Alfa et al., 2018)	Phase III
	ARG	CB-1158	Inhibiting arginase	Advanced and metastatic solid tumors (Steggerda et al., 2017)	Phase II
	ARG	nor-NOHA	Inhibiting arginase	Breast cancer (Secondini et al., 2017)	
	ARG	BEC hydrochloride	A boronic acid–based arginine analog and inhibiting ARG 1/2	Tumor-specific Th2 cells (Lorvik et al., 2016)	
Other amino acid metabolism	ASNS	[(bis-NHC)Pt(bt)]PF6 (1a)	A anticancer platinum(II) compound and targeting an active-site cysteine of ASNS	Colorectal cancer, liver cancer (Hu et al., 2019)	
	GSS	Polydatin	A bioactive ingredient and inhibiting GSS	Lung cancer (Chen et al., 2020)	
	IDH1	Ivosidenib	Inhibiting IDH1	Relapsed or refractory AML with a susceptible IDH1 mutation (Dhillon, 2018)	Approved
	IDH2	Enasidenib	Inhibiting IDH2	Relapsed or refractory AML with a susceptible IDH2 mutation (Kim, 2017)	Approved
	IDO1	Epacadostat	Inhibiting IDO1	Melanoma, lung cancer, solid tumors (Mitchell et al., 2018)	Phase I, II, and III
	IDO1	Navoximod	Inhibiting IDO1	Solid tumors (Nayak-Kapoor et al., 2018; Jung et al., 2019)	Phase I
Amino acid transporters	LAT1	KYT-0353	A novel tyrosine analog, inhibiting leucine uptake (Geier et al., 2013; Wang and Holst, 2015)	Colon cancer (Oda et al., 2010), oral cancer (Yun et al., 2014), T-cell lymphoblastic lymphoma (Rosilio et al., 2015), glioblastoma (Geier et al., 2013)	
	ASCT2, LAT1	GPNA	Inhibiting influx of leucine (Chiu et al., 2017); reducing glutamine uptake (Van Geldermalsen et al., 2016)	Non-small cell lung cancer (Hassanein et al., 2015), neuroblastoma (Ren et al., 2015), oligodendroglioma (Chiu et al., 2017), breast cancer (Van Geldermalsen et al., 2016)	
	System ${\text{X}}_{\text{c}}^{-}$	Erastin	A RAS-selective lethal compound, inducing ferroptosis	Osteosarcoma (Wang L. et al., 2020), diffuse large B cell lymphomas, RCC, AML (Lu B. et al., 2017)	

*GLS1, glutaminase1; BPTES, bis-2-(5-phenylacetamido-1,2,4-thiadiazol-2-yl)ethyl sulfide; PHGDH, phosphoglycerate dehydrogenase; SHMT1/2, serine hydroxymethyltransferase 1/2; MAT2A, methionine adenosine transferase 2A; RNR, ribonucleotide reductase; TYMS, thymidylate synthase; 5-FU, 5-fluoruoracil; DHFR, dihydrofolate reductase; MTHFR, methylenetetrahydrofolate reductase; ADI-PEG20, pegylated arginine deiminase; ARG, arginase; nor-NOHA, Nv-hydroxy-nor-Arginine; BEC hydrochloride, S-(2-boronoethyl)-L-cysteine hydrochloride; ASNS, asparagine synthetase; GSS, glutathione synthetase; IDH1, isocitrate dehydrogenase 1; AML, acute myeloid leukemia; IDH2, isocitrate dehydrogenase 2; IDO1, indoleamine 2, 3-dioxygenase 1; LAT1, L-type amino acid transporter 1; ASCT2, alanine/serine/cysteine-preferring transporter 2; GPNA, L-g-glutamyl-p-nitroanilide; RCC, renal cell carcinoma*.

Intracellular BCAAs are catabolized by highly reversible enzymes to provide nitrogen and carbon groups for the synthesis of biomass, energy production, nutritional signaling, and epigenetic regulation (Sivanand and Vander Heiden, 2020). Catabolism of BCAAs initiates at the transamination and transfer of nitrogen to α-KG by branched-chain amino transferases (BCATs) to produce glutamate and their respective branched-chain α-ketoacids (BCKAs) (Figure 1). There are two compartment-specific BCAA transaminases in humans, BCAT1 (or cBCAT), which is located in the cytoplasm and primarily expressed in the brain, and BCAT2 (or mBCAT), which is located in mitochondria with ubiquitous expression. They both efficiently and rapidly catalyze the reversible conversion of BCAAs and BCKAs. Thus, BCAAs and BCKAs likely exist in equilibrium in most cases. α-KG is an essential cofactor for α-KG-dependent dioxygenases and is required for DNA and histone demethylation (amino acids involved in epigenetic modification will be addressed in section Amino Acid Metabolism and Epigenetics). Thus, BCAT1 is an important metabolic enzyme for α-KG generation in acute myeloid leukemia (AML) progression (Raffel et al., 2017). Besides acute myeloid leukemia, BCAT1 is also activated in the blast crisis of chronic myelocytic leukemia (CML) (Hattori et al., 2017). Oncogenic RNA-binding protein musashi2 (MSI2) physically binds BCAT1 transcript and positively regulates its expression to drive CML progression (Hattori et al., 2017).

BCKAs can be further catabolized by the multimeric BCKA dehydrogenase enzyme complex (BCKDH) (Figure 1). It is to be noted that the BCKDH reaction is essentially irreversible in mammalian cells such that BCKA synthesis depends on essential amino acids, BCAAs, due to the lack of BCKAs *de novo* synthetases. Most amino acids are catabolized in the liver, except BCAAs. The liver has an active BCKDH to facilitate the consumption of BCKAs for gluconeogenesis or fatty acid synthesis (Neinast M. et al., 2019), but is deficient in BCAT, the initiator of BCAA breakdown. This may explain why BCAA levels in the urine or plasma have some predictive power for early-stage disease diagnosis (Mayers et al., 2014; Danai et al., 2018). In KRAS-mutant pancreatic ductal adenocarcinoma (PDAC), elevated levels of circulating plasma BCAAs present a 2-fold increased risk of future disease diagnosis (Mayers et al., 2014). The high circulating concentration of BCAAs may come from the breakdown of tissue protein that accompanies early-stage disease independent of both BCAT1 and BCAT2 (Mayers et al., 2014). However, p53 deletion-induced non-small cell lung carcinoma (NSCLC) is sensitive to the disruption of BCAT1/2 because the high expression of BCAA catabolic enzymes leads to active use of BCAAs (Mayers et al., 2016). Moreover, suppression of BCAA catabolic enzyme expression led to reduced cell proliferation and BCAA accumulation in liver carcinogenesis but not in regenerating liver tissues (Ericksen et al., 2019). BCKAs can be metabolized by BCKDH to branched-chain acyl-CoA, and blocking this step causes serious metabolic disorders, such as maple syrup urine disease (Xu et al., 2020) and propionic academia (Chen et al., 2017). Branched-chain acyl-CoA can be further metabolized in several steps to the TCA cycle, and intermediates, such as acetyl-CoA and/or succinyl-CoA, can be used for energy production (She et al., 2007) or acetylation modification (Campbell and Wellen, 2018).

BCAAs are important nutrient sources (Kamphorst et al., 2015). Leucine is the most abundant amino acid in proteins (Duan et al., 2016) and is usually administered orally or intravenously as a nutritional supplement to increase protein synthesis and maintain energy homeostasis for repairing muscle injuries. BCAAs and other essential nutrients, such as glucose and fatty acids, have strong metabolic crosstalk as cell signals for growth and stress responses (Nie et al., 2018). High BCAA levels inhibit glucose metabolism (Li T. et al., 2017), and, in turn, high glucose level inhibits BCAA degradation (Shao et al., 2018). Moreover, BCAA catabolism plays a positive functional role in adipocyte differentiation and lipogenesis (Green et al., 2016). Interruption of BCAA homeostasis by genetic or pharmacological inhibition of the BCKDH complex could affect insulin secretion and sensitivity (Shin et al., 2014; Bloomgarden, 2018; Zhou et al., 2019).

### Arginine Metabolism

Arginine is also categorized as a semi-essential or conditionally essential amino acid. It can be obtained from not only the *de novo* synthesis and turnover of proteins but also extracellular reservoirs like diet. Therefore, arginine deprivation is becoming a novel and promising clinical strategy for metabolism-based cancer therapy (Qiu et al., 2015; Xiong et al., 2016). Besides being used in protein synthesis, arginine serves as a precursor for polyamines, nitric oxide, creatine, and other amino acids. Arginine is taken up into the cell via the members of system y^+^, the cationic amino acid transporter (CAT) family, mainly CAT-1 (*SLC7A1*). In high L-arginine-dependent tumors, such as breast cancer (Abdelmagid et al., 2011), CRC (Lu et al., 2013), and HCC (Kishikawa et al., 2015), increased expression of the transporter CAT-1 (*SLC7A1*) has been observed, and CAT-1 (*SLC7A1*) knockdown decreases the viability of cancer cells and induces apoptosis. The liver-specific microRNA, miR122, is a direct negative regulator of CAT-1 (*SLC7A1*) (Kishikawa et al., 2015). Silencing of miR122 has been shown to upregulate CAT-1 (*SLC7A1*) expression, and subsequently increase arginine uptake to maintain large intracellular arginine pools for nitric oxide (NO) synthesis, resulting in increased resistance to the multi-kinase inhibitor sorafenib (Kishikawa et al., 2015). Another arginine transporter, CAT-2 (*SLC7A2*), is highly expressed in BRAF (v-raf murine sarcoma viral oncogene homolog B1) inhibitor-resistant melanoma, displaying metabolic reprogramming from glucose to arginine dependence (Li Y. Y. et al., 2017).

The *de novo* biosynthesis of arginine is carried out through the urea cycle, a process of ammonia detoxification by conversion to non-toxic urea. Carbamoyl phosphate, produced by carbamoyl phosphate synthetase 1 (CPS1), which is located in the mitochondria, together with ornithine is used to generate citrulline. Then, the rate-limiting enzyme in arginine synthesis, argininosuccinate synthetase 1 (ASS1), catalyzes the conversion of citrulline and aspartate into argininosuccinate, which is then cleaved into arginine and fumarate by argininosuccinate lyase (ASL) (Figure 1). The complete urea cycle only occurs in the liver for systemic waste nitrogen disposal. Outside the liver, some enzymes in the urea cycle have strong crosstalk with other metabolic pathways to rewire urea cycle intermediates to support the survival and proliferation of tumor cells (Keshet et al., 2018; Lee et al., 2018). Fumarate, also an intermediate metabolite of the TCA cycle, conventionally links arginine metabolism to glucose-generated energy metabolism. Specific alterations in the expression of the urea cycle enzymes in different cancers profoundly affect nitrogen-containing macromolecules, such as nucleotides and proteins, which are associated with tumor initiation and progression, even immunotherapy response (Lee et al., 2018). ASS1 expression has been reported to be decreased or even abolished in many cancers (Dillon et al., 2004), especially in malignant melanoma (Feun et al., 2012), HCC (Delage et al., 2010), and prostate cancer (Kim et al., 2009), which are thought of as arginine-auxotrophic cancers because of the inability to synthesize arginine and resulting susceptibility to arginine-deprivation therapy. A previous study demonstrated that in ASS1-deficient myxofibrosarcoma, ASS1 re-expression inhibited tumor growth by inhibiting tumor angiogenesis and inducing G1 phase arrest, indicating that ASS1 was a new tumor suppressor (Huang et al., 2013). ASS1 downregulation has been reported to be associated with advanced tumor stage, high local recurrence rate, and poor relapse- and metastasis-free survival (Nicholson et al., 2009; Lan et al., 2014). Arginine is cleaved into urea and ornithine by cytosolic arginase 1 (ARG1) or mitochondrial arginase 2 (ARG2), resulting in local L-arginine deprivation. The expression of arginases including ARG1 and ARG2 has been reported to be increased in cancers, such as gastric cancer, breast cancer, prostate cancer, colorectal cancer, and AML (Leu and Wang, 1992; Wu et al., 1996; Porembska et al., 2003; Mumenthaler et al., 2008; Mussai et al., 2013). Accumulation of ARG1 has been shown to drive polyamine production from the urea cycle (Lou et al., 2020).

Ornithine decarboxylase (ODC), the rate-limiting enzyme in polyamine biosynthesis and an oncogenic c-MYC transcriptional target (Bello-Fernandez et al., 1993), decarboxylates ornithine to form putrescine, the first mammalian polyamine. Interestingly, AMPK activates developmental Hedgehog signaling to promote polyamine biosynthesis in medulloblastoma by inducing translation of ODC (D'Amico et al., 2015), indicating that metabolism is a hub that connects development and cancer. Putrescine serves as an immediate precursor for the other two polyamines spermidine and spermine synthesis through receiving the aminopropyl from decarboxylated S-adenosylmethionine (dcAdoMet or decSAM) by spermidine synthase (SPDSY) and spermine synthase (SPMSY), successively (Figure 1). Polyamine anabolism, notably ODC, and polyamine catabolism are regulated by an oncogenic pathway. Spermidine/spermine N1-acetyltransferase 1 (SSAT) initiates polyamine catabolism, which decreases the cellular content of polyamines through transferring the acetyl group of acetyl-CoA to either spermidine or spermine, which can be oxidized by peroxisomal enzyme polyamine oxidase (PAOX). A recent study found that SAT1 (the gene encodes SSAT) is a transcriptional target of p53 and participates in p53-mediated ferroptosis (Ou et al., 2016). The increased expression of SAT1 promotes the depletion of spermidine and spermine, which leads to mitochondria-mediated apoptosis, and increases the sensitivity of drug-resistant cells to cisplatin-induced apoptosis (Mandal et al., 2015). Moreover, depletion of polyamines by SSAT significantly inhibits Wnt/β-catenin signaling for cell proliferation, migration, and invasion in HCC and CRC (Wang et al., 2017). Elevated polyamine levels are necessary for neoplastic transformation and tumor progression (Casero et al., 2018). With the rapid development of highly sensitive metabolite detection techniques, polyamines and polyamine metabolites in urine and plasma have become biomarkers used in the diagnosis and treatment response of various cancers (Casero et al., 2018).

Arginine and arginine-dependent polyamine production are important not only for biomass synthesis, which supports tumor aggressiveness, but also as regulators in immunometabolism (Mondanelli et al., 2017), which represents an important target for effective cancer immunotherapy. Methylation of arginine residues by protein arginine methyltransferases (PRMTs) has been investigated in cancer cells and immune cells. Currently, PRMT5 inhibition is a promising and effective therapeutic strategy (Guccione and Richard, 2019).

### Others

Recently, a metabolomics study involving 928 cell lines from over 20 cancer types identified 225 metabolites to profile the landscape of cancer metabolism (Li et al., 2019). Asparagine auxotrophy or asparagine addiction is also a common phenomenon in cancer research, especially in acute lymphoblastic leukemia (ALL) (Vettore et al., 2020). Elevated expression of asparagine synthetase (ASNS), which transfers an amide group from glutamine to aspartate for asparagine formation, is associated with resistance to asparaginase therapy in ALL (Lomelino et al., 2017). As a synergistic partner of glutamine, asparagine metabolism regulates tumor growth and metastasis (Luo et al., 2018). Decreased aspartate availability, due to inhibition of EAAT1 (*SLC1A3*) (${\text{system }}_{\text{A}{\text{G}}^{-}}$), an aspartate/glutamate transporter, has been suggested to increase the vulnerability of tumor cells under low oxygen (Garcia-Bermudez et al., 2018). Proline synthesis and proline cycling participate in redox signaling, and targeting proline metabolism may be an effective adjunct cancer therapy (Phang, 2019). Tryptophan and arginine have become effective targets of tumor immunotherapy (amino acids contributing to tumor immunity are addressed in section Amino Acid Metabolism and Tumor Immunity). Methionine uptake and metabolism is a nexus of epigenetic regulation, redox maintenance, and metabolism of other amino acids (Sanderson et al., 2019) (Figure 1), which hints at diet-directing cancer therapies. The anti-folate–anti-cancer chemotherapeutic drug methotrexate (Table 1) is used clinically for curing multiple cancers, especially acute leukemia, despite its serious side effects. Kanarek et al. found that histidine catabolism affects the sensitivity of cells to methotrexate, and dietary supplementation of histidine maintains a suitable chemotherapy effect with a low dose of medication. This highlights the importance of amino acid dietary supplements in the treatment of some cancers (Frezza, 2018; Kanarek et al., 2018). Recently, a comprehensive review has summarized the effect of various dietary modifications on the efficacy of cancer therapies, indicating the potential of future combinations of diets and therapies for specific patients (Kanarek et al., 2020).

## Amino Acid Metabolism and mTORC1 Signal

The evolutionarily conserved atypical serine/threonine kinase mTOR, which belongs to the PI3K-related kinase (PI3KK) superfamily, is the convergence point of anabolic and catabolic processes. It senses fluctuations in extracellular and intracellular amino acids to modulate cellular growth, metabolism, and survival (Figure 2). The constitutive heterodimeric Rag GTPases, which consist of GTP-loaded RagA/B and GDP-loaded RagC/D, promote the translocation of mTORC1 to the lysosome, where it physically interacts with and then is activated by Rheb (Wolfson and Sabatini, 2017). Regulation of the RAG GTPases, and hence mTORC1 activity, by amino acid levels is achieved through specific amino acid sensor proteins. Sestrins were the first identified amino acid sensors in response to cytosolic leucine levels. As negative regulators of the mTORC1 pathway (Budanov and Karin, 2008), sestrins bind and inhibit GATOR2 (Parmigiani et al., 2014), a positive regulator for nutrient-sensing of this pathway, under leucine deprivation. Sestrins repress mTORC1 signaling through an AMPK-TSC2-dependent (Budanov and Karin, 2008) or -independent (Peng et al., 2014) manner. Knockout of sestrins, or at least impairment of their leucine-binding ability, could make mTORC1 activity insensitive to leucine stimulation. Surprisingly, reports have shown that leucyl-tRNA synthetase (LARS) is also an intracellular leucine sensor. LARS, as a GTPase-activating protein (GAP), interacts with Rag GTPase (Han et al., 2012) or directly mediates leucylation of RagA/B (He et al., 2018), to activate mTORC1. CASTOR1/2, another GATOR2-binding protein in arginine-depleted conditions, has been validated as a cytosolic arginine sensor (Chantranupong et al., 2016). CASTOR1/2 can form either a CASTOR1 homodimer or CASTOR1/2 heterodimer to inhibit mTORC1 activity. Intracellular methionine is sensed as SAM by SAMTOR (Gu et al., 2017). Unlike leucine and arginine, SAMTOR directly binds and activates GATOR1 but does not inhibit GATOR2, which is upstream of GATOR1 in this pathway. Reduced SAM levels due to methionine starvation promote the dissociation of SAM with SAMTOR and then promote the association of SAMTOR with GATOR1 to repress the mTORC1 pathway. Leucine-, arginine-, and methionine-derived SAM can bind their sensors during high intracellular concentrations, thereby disrupting the interaction of the sensors with GATOR1/2, relieving the inhibition of GATOR complex, and activating mTORC1.

Aside from cytosolic amino acid sensors, intra-lysosomal amino acid sensing coincided with the identification of SLC38A9, a lysosomal transmembrane protein likely belonging to arginine transporters and an integral part of mTORC1 activation via Ragulator-RAG GTPases (Jung et al., 2015; Rebsamen et al., 2015; Wang S. et al., 2015). Notably, SLC38A9 is necessary for not only leucine but also other essential amino acids (e.g., glutamine, tyrosine, and phenylalanine) that are generated via lysosomal proteolysis and efflux from lysosomes in an arginine-stimulated fashion to activate mTORC1 (Rebsamen et al., 2015; Goberdhan et al., 2016; Wyant et al., 2017). This is important for pancreatic cancer cells, which take full advantage of macropinocytosed protein as a nutrient source for tumorigenesis (Wyant et al., 2017). In pancreatic KRAS-mutated cancer cells, which can proliferate using albumin as the extracellular source of leucine, loss of SLC38A9 or its transport function strongly inhibited mTORC1 activation by macropinocytosed albumin as evidenced by cell proliferation and tumor formation (Goberdhan et al., 2016; Wyant et al., 2017). Glutamine activates mTORC1 by enhancing glutaminolysis and the production of α-KG, which is helpful for the GTP-loading of RagB and lysosomal translocation (Duran et al., 2012). In particular, glutamine can also promote mTORC1 translocation to the lysosome and activate it in a RAG-independent manner (Stracka et al., 2014; Jewell et al., 2015) via the small GTPase ADP-ribosylation factor 1 (ARF1) (Jewell et al., 2015). Amino acids as nutrients, in particular leucine, arginine, and glutamine, are the most effective activators of mTORC1. mTORC1 activation via stimulation of different amino acids helps rapidly proliferating cancer cells survive under genotoxic, oxidative, and nutritional stress.

High levels of intracellular amino acids activate mTORC1, and, in turn, mTORC1 activation regulates amino acid availability through downstream signaling effectors (Figure 2). mTORC1 directly phosphorylates ribosomal protein S6 kinase 1 (S6K1) and/or eukaryotic translation initiation factor 4E-binding protein 1 (4EBP1) to increase translation, including translation of metabolic enzymes and metabolism-related transcription factors (Saxton and Sabatini, 2017). mTORC1, via S6K1, also improves the translation of c-MYC and, in turn, inhibits the transcription of miR-23a and miR-23b, which post-transcriptionally repress GLS (Gao et al., 2009). Therefore, mTORC1 upregulates the expression of GLS and promotes glutaminolysis. mTORC1 also promotes glutamine breakdown by preventing SIRT4-mediated ADP-ribosylated GDH inhibition (Csibi et al., 2013). This is because cAMP-responsive element-binding 2 (CREB2), which transcriptionally regulates SIRT4, would be induced to degrade by the proteasome during mTORC1 activation. Through phosphorylation inhibition of 4E-BP1, mTORC1 can increase ODC translation and promote polyamine synthesis (Rousseau et al., 1996). c-MYC induces ASS1 expression by directly binding its promoter and increasing the intracellular arginine capacity (Tsai et al., 2012). In addition, ODC is another transcriptional target of c-MYC, and activation of c-MYC increases ODC expression (Bello-Fernandez et al., 1993). Mitochondrial serine hydroxymethyltransferase (SHMT2) catalyzes serine and THF to form glycine and 5, 10-methylene-THF. Methylene-THF dehydrogenase 2 (MTHFD2) is subsequently responsible for the production of 10-formyl-THF, which is mostly exported and regenerated in the cytosol to participate in purine biosynthesis (Yang and Vousden, 2016). In response to growth signals, mTORC1 activates ATF4, which stimulates the expression of core enzymes in serine synthesis and the folate cycle for *de novo* purine synthesis required for DNA replication and ribosome biogenesis in rapidly growing and proliferating tumor cells (Denicola et al., 2015; Ben-Sahra et al., 2016). ATF4 also transcriptionally regulates a series of amino acid transporters, to mediate amino acid uptake (Zhu and Thompson, 2019). On metabolic stress, ASCT2 (*SLC1A5*), LAT1 (*SLC7A5*), CAT1 (*SLC7A1*), GLYT1 (*SLC6A9*), and xCT, the light chain subunit of system ${\text{X}}_{\text{c}}^{-}$ (SLC7A11), are elevated by ATF4 activation, which facilitates the uptake of glutamine, leucine, arginine, lysine, glycine, and cystine (Lopez et al., 2007; Koppula et al., 2017; Yue et al., 2017; Pathria et al., 2018; Zhang et al., 2018, 2019; Augusto et al., 2019; Edick et al., 2020). Besides modulating the anabolism and catabolism of certain amino acids, mTORC1 also controls their uptake into the cytoplasm by inducing and sustaining the cell surface expression of amino acid transporters. In lymphoma cells, rapamycin-induced mTORC1 inhibition reduces the expression of a variety of amino acid transporters, including LAT1 (*SLC7A5*) (Peng et al., 2002).

Once mutant and/or misfolded proteins with a loss- or gain-of-function or damaged and potentially harmful cellular structures cannot be effectively eliminated, cells undergo malignant transformation, which may progress to carcinogenesis (Edinger and Thompson, 2003). Viewed from this perspective, mTORC1 is a pro-oncogenic factor as it represses autophagy by either directly inhibiting multiple stages of autophagy or indirectly regulating lysosomal biogenesis (Rabanal-Ruiz et al., 2017) (Figure 2). mTORC1 directly phosphorylates and inactivates the autophagy-initiating kinase ULK1 (Jung et al., 2009; Kim et al., 2011), ULK1-associated ATG13 (Jung et al., 2009), and ATG14 (Yuan et al., 2013), which disrupts autophagy induction and autophagosome nucleation and maturation. mTORC1 also phosphorylates TFEB (Martina et al., 2012; Settembre et al., 2012), a master transcription factor coordinating lysosomal biogenesis and autophagic gene expression. Pharmacological inhibition of mTORC1 or starvation promotes TFEB nuclear localization and its transcriptional activity (Martina et al., 2012; Settembre et al., 2012). Tumors, especially solid tumors, are often in a nutrient-limited environment. When amino acids are scarce, mTORC1 senses the fluctuations in amino acid levels and can be suppressed to increase amino acid availability from protein turnover via autophagy for tumor growth. Then, once amino acids are available in sufficient quantity, mTORC1 is reactivated, which inhibits autophagy. In cancer cell lines, the mTORC1 signal can also promote *de novo* lipid synthesis through the sterol responsive element-binding protein (SREBP) transcription factors, which induce the expression of metabolic genes responsible for lipid biosynthesis, including fatty acid synthase (FASN) (Duvel et al., 2010). High expression and activation of SREBPs have been reported in various cancers and have been shown to promote tumor growth (Cheng et al., 2018). Moreover, N-glycosylation of SREBP cleavage-activating protein (SCAP) is essential for Golgi localization and activation of SREBP once EGFR induced glucose uptake in glioblastoma multiforme (GBM) (Cheng et al., 2015, 2016). In all, mTORC1 signals serve as a central hub of amino acid sensing and a variety of metabolic regulatory pathways, including proteins, nucleotides, and lipids synthesis, as well as other anabolic or catabolic processes (Ben-Sahra and Manning, 2017).

## Amino Acid Metabolism and Epigenetics

Epigenetic modification can regulate gene expression by activating or inhibiting gene transcription without changing the DNA sequence, ultimately affecting embryonic development, stem cell differentiation, senescence, and tumorigenesis (Brien et al., 2016; Cavalli and Heard, 2019). Epigenetic aberrations, especially DNA methylation, histone modifications, chromatin remodeling, and small RNAs, have been described in malignant hematological and solid tumors and can be considered as common features of cancer development and progression (Sharma and Rando, 2017; Toh et al., 2017; Nebbioso et al., 2018). Tumorigenesis-associated metabolic reprogramming affects the genomic status by regulating the enzymes for epigenetic modifications, which commonly utilize key metabolites as either substrates or allosteric regulators (Etchegaray and Mostoslavsky, 2016; Van Der Knaap and Verrijzer, 2016; Sabari et al., 2017). The chemical modification of DNA and histones is very sensitive to cell metabolism and nutritional status (Su et al., 2016).

DNA methylation refers to the transfer of the methyl group provided by SAM to the 5-position carbon atom of cytosine, catalyzed by methyltransferase (DNMT), to form 5′-methylcytosine. During tumorigenesis, abnormal hypermethylation of the cytosine in CpG islands and hypomethylation of the whole genome result in genome instability and alterations in gene expression profile, including silencing of tumor suppressor genes, endogenous retro-elements, and tumor antigens, and activation of oncogenes (Liang and Weisenberger, 2017; Schorn et al., 2017). Intracellular SAM, the one carbon-derived methyl donor, is synthesized by methionine and ATP in the presence of methionine adenosine transferase (Figure 1). As the main methyl donor in cells, SAM also mediates a variety of methylation reactions, aside from DNA methylation, including histone, RNA, and some protein amino acid residue methylation (Teperino et al., 2010). Uptake and metabolism of folate, vitamins B_6_ and B_12_, choline, betaine, serine, and glycine may influence the methyl donor pool, and, ultimately, degrees of methylation modifications (Sapienza and Issa, 2016). LAT1 (*SLC7A5*) is responsible for inputting essential amino acids, including methionine; therefore, LAT1 (*SLC7A5*) is essential for maintaining the intracellular SAM concentration. The expression of LAT1 is upregulated in many cancers and is associated with poor prognosis (Yanagisawa et al., 2012; Isoda et al., 2014; Shimizu et al., 2015). Downregulation of LAT1 (*SLC7A5*) suppresses methionine input, thus reducing the level of cellular SAM, resulting in methylation depletion of some histones and inhibition of tumor growth. More importantly, the downregulation of the EZH2 gene leads to a decrease in LAT1 (*SLC7A5*) expression, and, in turn, the downregulation of LAT1 (*SLC7A5*) or the depletion of essential amino acids can also induce a decrease in EZH2 expression (Dann et al., 2015). The positive feedback loop of EZH2-LAT1 (*SLC7A5*) indicates the potential of LAT1 (*SLC7A5*) as a target for cancer therapy (Hafliger and Charles, 2019).

In addition to regulating epigenetic methylase activity, metabolism also affects epigenetic enzymes involved in demethylation in cancer cells. Through α-KG-dependent dioxygenase, the amino acid metabolite α-KG is also involved in regulating histone and DNA demethylation (Xu et al., 2011; Xiong et al., 2018; Lio et al., 2019). These α-KG-dependent dioxygenases include the Tet family, which catalyzes the conversion of 5-methylcytosine to 5-hydroxymethylcytosine; the Jumonji C domain-containing histone demethylase, which catalyzes the demethylation of mono-, bi-, and trimethyl lysine residues by oxidation; and the prolyl hydroxylase (PHD) family, which hydroxylates hypoxia-inducible factor (HIF) to mediate its degradation (Wu et al., 2018; Duan et al., 2019; Lio et al., 2019). These reactions require the participation of α-KG, so low levels of α-KG may cause hypermethylation of DNA and histones. IDH mutations were first reported in GBM and were later found in other tumors, such as AML, cholangiocarcinoma, and chondrosarcoma (Parsons et al., 2008; Marcucci et al., 2010; Amary et al., 2011; Borger et al., 2012). Normal IDH catalyzes the dehydrogenation of isocitrate to α-KG. However, when it mutates, IDH converts α-KG to 2-hydroxyglutaric acid (2-HG) and competitively inhibits α-KG-dependent DNA and histone demethylases (Xu et al., 2011), leading to a hypermethylation phenotype and may alter the differentiation of cancer stem cells (Yang et al., 2012; Tommasini-Ghelfi et al., 2019). In IDH1 mutant glioblastoma, BCAT1 is transcriptionally suppressed because of the hypermethylation of three CpGs in the promoter, and this is also the consequence of IDH1 mutation-induced 2-hydroxyglutarate (2-HG) (Tonjes et al., 2013). The suppression of BCAT1 can block glutamate excretion and, thus, leads to reduced growth and invasiveness of glioblastoma (Tonjes et al., 2013). In human AML stem cells, BCAT1 is overexpressed, and the BCAA pathway is activated by the low levels of α-KG, displaying a DNA hypermethylation phenotype similar to IDH mutant-positive cancers (Raffel et al., 2017). Knockdown of BCAT1 causes accumulation of α-KG, promoting EGLN1-mediated HIF1α protein degradation and leukemia-initiating arrest (Raffel et al., 2017). For patients with IDH (WT)/TET2 (WT) myeloid leukemia, a high level of BCAT1 is a strong predictor of worse survival outcomes, and the BCAT1 level significantly increases on disease relapse (Raffel et al., 2017). In recent years, IDH inhibitors targeting IDH mutants, including ivosidenib and enasidenib (Table 1), have been approved by the Food and Drug Administration to be used in patients with IDH1 or IDH2 mutant recurrent or refractory AML, respectively (Kim, 2017; Dhillon, 2018), while trials of IDH inhibitors for other tumors such as cholangiocarcinoma, chondrosarcoma, and myelodysplastic syndrome are still underway (Abou-Alfa et al., 2020; Stein et al., 2020; Tap et al., 2020). Unlike α-KG, abnormal accumulation of succinate and fumarate in tumor tissues represses PHD, thus reducing HIF1 α hydrolysis, suppressing demethylation of DNA and histones, and promoting the occurrence and development of tumors (Cavalli and Heard, 2019). The mutation or decrease in the succinate dehydrogenase (SDH) gene leads to an increase in the concentration of succinate. SDH gene mutations have been confirmed to exist in many tumors, such as gastrointestinal stromal tumors, renal cell carcinoma, pheochromocytoma, and paraganglioma (Pasini and Stratakis, 2009; Dwight et al., 2013; Calio et al., 2017).

Histone post-translational modifications are another set of epigenetic marks in cancers (Audia and Campbell, 2016). Emerging evidence suggests that eight types of Lys acylations on histones affect chromatin structural changes and gene expression (Sabari et al., 2017). Histone acetylation, which is well characterized, is controlled by the opposing activities of histone acetyltransferases (HATs) and histone deacetylases (HDACs). HATs catalyze the addition of acetyl groups from the acyl donor acetyl-CoA, which can be produced by glucose, fatty acids, and BCAA metabolism (Figure 1), to lysine residues in histone tails—thus called histone acetylation. Histone acetylation can be regulated by acetyl-CoA from different sources in different cell conditions (Sivanand et al., 2018). HDACs, responsible for the removal of acetyl groups from histone lysine residues, have been reported to have abnormal expression in cancers, and HDAC inhibitors (HDACi) have been considered as potential drugs in cancer treatment (Audia and Campbell, 2016; Li and Seto, 2016; Peleg et al., 2016; San Jose-Eneriz et al., 2019; Mirzaei et al., 2020; Wang P. et al., 2020; Wang X. et al., 2020).

The metabolic reprogramming of cancer cells through amino acids affects epigenetic change. In turn, epigenetic modifications in key enzymes in amino acid metabolism induce the malignant transformation of cells (Blanc and Richard, 2017; Ali et al., 2018). A recent study has shown that argininosuccinate synthase 1 (ASS1) and spermidine/spermine N1-acetyltransferase (SAT1), the central enzymes for arginine metabolism, are hypermethylated in cisplatin-resistant bladder cancer cells (Yeon et al., 2018). Downregulation of ASS1 caused by promoter methylation increases the susceptibility of tumor cells to PEGylated arginine deiminase (ADI-PEG20) (Table 1), a drug for arginine-deprivation treatment that has been used in clinical trials for a variety of tumors (Delage et al., 2012; Syed et al., 2013; Mcalpine et al., 2014). Another recent screening identified that H3K9 demethylation-mediated upregulation of BCAT1 and subsequent BCAA metabolic reprogramming is able to enhance the capacity for sublethal epidermal growth factor receptor (EGFR) inhibitor (TKI) resistance by producing ROS scavengers in lung cancer, which is a cancer where EGFR mutations are commonly found (Wang et al., 2019b).

## Amino Acid Metabolism and Tumor Immunity

The tumor microenvironment is mainly composed of tumor cells, immune cells, stromal cells, extracellular matrix, as well as various cytokines and chemokines (Clara et al., 2020; Galon and Bruni, 2020). Metabolic crosstalk within the tumor is very important to maintain the progression of the tumor. In pancreatic ductal adenocarcinoma, matrix-related pancreatic stellate cells become the nutritional source of cancer cell growth and metabolism by secreting non-essential amino acids (Sousa et al., 2016). Stromal fibroblasts provide a nitrogen source for the proliferation of epithelial tumors and maintain the activity of cancer cells through the production of asparagine (Linares et al., 2017). In turn, cancer cell-derived glutamate supports the glutathione pathway in fibroblasts to balance the redox state (Bertero et al., 2019). In addition to the tumor matrix, a complex interplay between immune cells and tumor amino acid metabolism is emerging. To respond to antigen signals and cytokines, the upregulation of amino acid transporters is used to meet the demand for the supply of large neutral amino acids in activating immune cells (Sinclair et al., 2013). Extracellular alanine, as an environmental nutrient, is also required during T-cell activation and memory T-cell restimulation (Ron-Harel et al., 2019). However, tumor cells often compete with normal cells, including immune cells, for nutrients in the tumor microenvironment (Buck et al., 2017). Tumor cells compete for extracellular resources, limit the amino acid supply to immune cells, and inhibit immune cell functions as a means to evade the immune system. At the same time, amino acid catabolism, particularly L-arginine and tryptophan, also has important effects on the anti-tumor immune response.

L-Arginine is a multifunctional amino acid. High levels of intracellular L-arginine have been shown to reprogram the metabolism in activated T cells from glycolysis to oxidative phosphorylation, promoting the production of central memory-like T cells with anti-tumor activity and increasing the survival of T cells (Geiger et al., 2016). On the contrary, arginine depletion blocks T cells in the G0–G1 phase, resulting in reduced proliferation and functional impairment (Oberlies et al., 2009; Garcia-Navas et al., 2012). Myeloid-derived suppressor cells (MDSCs) and activated tumor-associated macrophages express ARG1, which consumes arginine to form a microenvironment that is not conducive to T cells, resulting in tumor immunosuppression (Rodriguez et al., 2017; Sawa-Wejksza and Kandefer-Szerszen, 2018). In ovarian cancer, ARG1, using extracellular vesicles as a carrier, inhibits T-cell proliferation, which is beneficial to tumor growth and immune escape (Czystowska-Kuzmicz et al., 2019). In AML, blasts secrete ARG2 to form a high plasma concentration of ARG2, which inhibits the proliferation of T cells and hematopoietic progenitor cells and enhances the inhibitory microenvironment. Small molecule inhibitors of ARG2 have been shown to upregulate the immune activity and almost completely counteract the immunosuppression, suggesting that ARG2 can be used as a target for the immunotherapy of AML (Mussai et al., 2013). Multiple ARG inhibitors are being developed in tumor immunotherapy. The ARG1 small molecule inhibitor CB-1158 (Table 1) blocks arginase, attenuates myeloid cell–mediated immune escape and tumor growth, and is beneficial to T-cell proliferation (Steggerda et al., 2017). nor-NOHA (Table 1) weakens the inhibition of MDSC on T-cell proliferation and tumor cell metastasis in breast cancer (Secondini et al., 2017). NOHA inhibits the death of T cells and restores the activity of cytotoxic T lymphocytes in prostate cancer (Bronte et al., 2005). 6-Gingerol is used as an arginase inhibitor to reprogram tumor-supported macrophages to ameliorate lung carcinogenesis in the uratan-induced lung cancer model (Yao et al., 2018). Furthermore, BEC is a slow-binding inhibitor of arginase, which is still in the preclinical stage (Pudlo et al., 2017).

Tryptophan is one of the essential amino acids in the human body and is decomposed mainly through the kynurenine pathway, in addition to producing physiologically active substances, such as 5-hydroxytryptamine and melatonin. Kynurenine, a metabolite of tryptophan, upregulates T-cell PD-1 levels by inducing and activating the aryl hydrocarbon receptor (AhR), while AhR upregulates transporters to promote T cells to uptake kynurenine, forming a positive feedback loop and inhibiting anti-tumor immune responses (Liu et al., 2018). In addition, tryptophan depletion and kynurenine accumulation induce the formation of immunosuppressive regulatory T cells (Tregs), leading to tumor growth (Heng et al., 2016). Indoleamine 2, 3-dioxygenase 1 (IDO1), indoleamine 2, 3-dioxygenase 2 (IDO2), and tryptophan-2, 3-dioxygenase (TDO) catalyze the rate-limiting step of tryptophan catabolism in the kynurenine pathway to consume tryptophan and produce kynurenine. IDO1 has been shown to be highly expressed in many cancers and is associated with poor prognosis (Folgiero et al., 2014; Feng et al., 2020). At the same time, in addition to being expressed in tumor cells, IDO is also expressed in stromal cells, such as fibroblasts and vascular endothelial cells (Haniffa et al., 2007; Riesenberg et al., 2007). Interestingly, in renal clear cell carcinoma, IDO expressed by neovascularization endothelial cells is an inhibitory factor in tumor growth because it limits the flow of tryptophan from the blood to the tumor or produces tumor toxic substances (Riesenberg et al., 2007). New evidence also shows that TDO is overexpressed in uveal melanoma and breast cancer, and correlates with a poor prognosis in breast cancer, which suggests that TDO may be a new target for cancer immunotherapy (Liu et al., 2020; Terai et al., 2020). However, there is minimal research regarding IDO2 at present. IDO1 inhibitors have been tested in a variety of anti-cancer treatments, including epacadostat (Table 1), indoximod, and navoximod, but compared with the results of preclinical trials, the efficacy of IDO1 inhibitors alone have not been objectively reflected in the clinical trials (Soliman et al., 2016; Beatty et al., 2017; Nayak-Kapoor et al., 2018). Therefore, studies on IDO1 inhibitors have shifted to studies in combination with other drugs. In the ECHO-202/KEYNOTE-037 trial, epacadostat combined with the PD-1 inhibitor perbrolizumab showed extensive anti-tumor activity against a variety of cancer types (Mitchell et al., 2018). However, in another study, epacadostat combined with pembrolizumab did not improve the prognosis of patients with melanoma (Long et al., 2019). Moreover, the phase I study of navoximod combined with the PD-L1 inhibitor, atezolizumab, also showed no significant benefit in the addition of navoximod (Jung et al., 2019).

## Amino Acid Metabolism, ROS, and Ferroptosis

Ferroptosis is a type of programmed cell death, which differs from classic caspase-dependent apoptosis driven by iron-catalyzed lipid peroxidation (Dixon et al., 2012; Yang and Stockwell, 2016; Stockwell et al., 2017). Special morphological changes that occur in the cell during ferroptosis include mitochondrial contraction and increased mitochondrial membrane density. Furthermore, various mitochondrial genes and metabolism both play an important role in this process (Dixon et al., 2012; Gao et al., 2019). Ferroptosis plays an important role in cell metabolism, redox status, degenerative diseases, and cancer (Stockwell et al., 2017). Polyunsaturated fatty acids (PUFAs) on the cell membrane can be oxidized by one or more lipoxygenases (LOXs), which contain di-iron active sites to form lipid peroxides (L-OOH). In the presence of iron, lipid peroxides turn into toxic lipid free radicals (L-ROS), which leads to the fragmentation of polyunsaturated fatty acids incorporated into phospholipids and cell death (Dixon, 2017). The phospholipid peroxidase glutathione peroxidase 4 (GPX4), a GSH-dependent lipid repair enzyme, can prevent the development of this process by eliminating lipid peroxides. Ferroptosis induction by inhibiting GPX4 is expected to become a most promising therapeutic strategy for promoting cancer cell death and treating some cancers (Dixon and Stockwell, 2019). However, it should be noted that the response of GPX4 inhibitors in different cancer cell lines is not consistent (Zou et al., 2019). Two studies from two different institutions recently identified a novel iron death signaling pathway independent of GPX4 (Bersuker et al., 2019; Doll et al., 2019). They both showed that overexpression of ferroptosis suppressor protein 1(FSP1), which was initially named apoptosis-inducing factor mitochondrial 2 (AIFM2), could protect cells from ferroptotic death. This is the first time that an enzyme catalytic system has been found to compensate for GPX4 deficiency in ferroptosis. Myristoylation of FSP1 is crucial for the inhibition of ferroptosis (Bersuker et al., 2019; Doll et al., 2019) because it reduces CoQ10 to prevent lipid oxidation (Bersuker et al., 2019).

As an essential intracellular antioxidant, reduced glutathione (γ-L-glutamyl-L cysteinylglycine, GSH) synthesis requires the cystine/glutamate antiporter system ${\text{X}}_{\text{c}}^{-}$ (encoded by *SLC7A11*) to import cystine from the environment and reduce it to cysteine. Consequently, an extracellular cystine or intracellular cysteine deficiency or inhibition of system ${\text{X}}_{\text{c}}^{-}$ (*SLC7A11*) would reduce the GSH level, inhibit GSH-dependent GPX4 activity, and lead to the intracellular accumulation of L-ROS and cell death (Dolma et al., 2003; Hayano et al., 2016; Stockwell et al., 2017). Cystine depletion can induce ferroptosis. Extracellular cystine deprivation increases ROS generation and inhibits NADPH oxidase 4 (NOX4), which inhibits ferroptosis by antioxidation, therefore inducing cell death in human mammary epithelial (HME) cells harboring an EGFR mutation (Poursaitidis et al., 2017). Cystine deficiency can also mitigate the tumor growth in EGFR mutated NSCLC xenografts (Poursaitidis et al., 2017). In head and neck cancer (HNC), dihydrolipoamide dehydrogenase (DLD) gene silencing blocked cystine deprivation-induced ferroptosis, displaying decreased lipid ROS and mitochondrial iron levels (Shin et al., 2020). Intracellular cysteine can be synthesized from methionine by the transsulfuration pathway (Figure 1) and not only by the reduction of imported cystine. An unbiased genome-wide siRNA screen for suppressors of ferroptosis found that loss of cysteinyl-tRNA synthetase (CARS) increases cystathionine and activates the transsulfuration pathway to suppress ferroptosis, which is induced by the well-known system ${\text{X}}_{\text{c}}^{-}$ (*SLC7A11*) inhibitor, erastin (Table 1) (Hayano et al., 2016). This resistance to ferroptosis decreases L-ROS but not iron levels (Hayano et al., 2016). Erastin and its analogs, sulfasalazine and sorafenib, as small molecule inhibitors of system ${\text{X}}_{\text{c}}^{-}$ (*SLC7A11*), have been developed. Small molecule–induced ferroptosis strongly inhibits tumor growth and may be a promising therapeutic modulator for enhancing the sensitivity of chemotherapeutic drugs, especially as drug resistance is emerging (Lu B. et al., 2017). High extracellular concentrations of glutamate, another raw material for glutathione synthesis, can inhibit system ${\text{X}}_{\text{c}}^{-}$ (*SLC7A11*) and induce iron-dependent non-apoptotic cell death, which may explain the toxic effect of excessive glutamate accumulation in the nervous system (Yang and Stockwell, 2016; Angeli et al., 2017). However, mitochondrial glutaminase, GLS2, instead of GLS1, has been shown to be required for ferroptosis, although both enzymes catalyze glutamine to glutamate (Gao et al., 2015b). p53 transcriptionally activates the expression of GLS2 (Jennis et al., 2016) and SAT1 (Ou et al., 2016) (a polyamine catabolic enzyme) and transcriptionally represses *SLC7A11* (Jiang et al., 2015). In addition, suppressor of cytokine signal transduction protein 1 (SOCS1) is required for p53 activation and regulates the expression of p53 target gene for cellular senescence. SOCS1 can reduce the expression of SLC7A11, in addition to GSH levels, sensitizing cells to ferroptosis (Saint-Germain et al., 2017). Furthermore, glutamine catabolism, known as glutaminolysis, provides fuel for the TCA cycle, which is essential for the induction of ferroptotic cell death (Gao et al., 2015a,b). When glutaminolysis is obstructed, both cystine starvation and erastin treatment fail to trigger ROS accumulation, lipid peroxidation, and subsequent ferroptosis (Gao et al., 2015b; Stockwell et al., 2017). Glycine, an important one-carbon source, is the third amino acid added to γ-glutamylcysteine, the condensation product of glutamate and cysteine, to form glutathione (Figure 1). Glycine deficiency decreases the synthesis of glutathione and promotes the production of ROS (Locasale, 2013; Yang and Vousden, 2016; Chen et al., 2018; Zhuang et al., 2019). Decreased GSH and accumulated ROS levels lead to lipid peroxidation and ferroptosis-mediated tumor suppression.

## Conclusions and Future Perspectives

In addition to essential amino acids, many non-essential amino acids and semi-essential amino acids also need an extracellular supply. Therefore, amino acid metabolism plays an important role in maintaining tumor proliferation and homeostasis. Amino acids are essential nutrition and energy sources for cancer cell growth and, as intermediates, connect glucose, lipid, and nucleotide metabolism. The intermediate metabolites of glutamine can enter the TCA cycle, thus ensuring the replenishment of the TCA cycle under the condition of glucose deficiency. Serine is derived from glycolysis, which produces glycine and contributes methyl groups to ensure the one-carbon cycle. Glutamine and BCAA catabolism control α-KG-dependent DNA and histone demethylation. Arginine and tryptophan participate in the regulation of T-cell survival, proliferation, and activation, becoming an effective target of tumor immunotherapy. Cystine and cysteine deficiency decrease intracellular antioxidant GSH, increase L-ROS, and induce ferroptotic death. Most amino acids can activate mTORC1 and, in turn, are regulated by the mTORC1 signaling pathway. Moreover, many amino acid transporters also participate in the mTOR signal transduction pathways, which make the relationship between amino acids and mTOR more complex.

As discussed previously, most studies regarding tumor amino acid metabolism focus on the regulatory mechanism, which contributes to tumor growth (as an essential nutrition, energy source, or raw materials), epigenetic modification, tumor immunity, and ferroptosis. Based on these discoveries, many novel strategies for cancer therapy have been developed. However, until now, few drugs have been used in clinical cases (Table 1), which is due to the heterogeneity of tumors and their complex regulatory mechanisms. The early cancer therapies referred to antimetabolites, such as methotrexate (a folate analog) and 5-FU (a pyrimidine analog), that competitively inhibit key metabolic enzymes (e.g., dihydrofolate reductase and thymidylate synthase) in *in vivo*, thereby affecting or antagonizing cancer cell metabolism and proliferation (Lukey et al., 2017). Targeting the high-expression enzymes in cancer amino acid metabolism via small molecule inhibitors or RNAi-targeting approaches is emerging as a crucial therapy (Martinez-Outschoorn et al., 2017; Secondini et al., 2017; Jones et al., 2018; Maggi and Scotti, 2019). However, many cancers exhibit a high demand for specific amino acids from exogenous supplies or endogenous release; thus, specific amino acid deprivation by drugs (e.g., apilimod, DQ661, and bafilomycin A1), which shut down nutrient scavenging pathways (e.g., suppressing lysosomal fusion, acidification, and nutrient export from lysosome), have aroused great interest (Gayle et al., 2017; Lukey et al., 2017; Finicle et al., 2018; Tabe et al., 2019). Blocking of amino acid uptake by pharmacologically inhibiting relevant transporters also disrupts tumorous metabolic addictions (Lukey et al., 2017; Jones et al., 2018; Hafliger and Charles, 2019). Recent developments have begun to shed light on combination treatments with other therapies, such as chemotherapy, radiation therapy, immunotherapy, or targeted therapy (Secondini et al., 2017; Keshet et al., 2018; Ramapriyan et al., 2019). Immune activation by interferon gamma (IFNγ) releasing from CD8 (+) T cells downregulates the expression of system ${\text{X}}_{{\text{c}}^{-}}$, which induces ferroptosis. So, enhanced ferroptosis of tumor cells in combination with checkpoint blockade immunotherapy is a new therapeutic strategy (Wang W. et al., 2019). Some researchers have also provided information on various dietary modifications as cancer therapies, which may improve the current treatments (Kanarek et al., 2018, 2020). Testing these hypotheses is an exciting direction, but further work needs to be completed. In addition to cancer treatment, cancer prevention and non-invasive diagnosis techniques have gained attention, for example, restricting certain amino acids from the diet or dietary modifications to combat the growth and reproduction of the cancerous cells. Nonetheless, there remains an urgent need for novel potential and effective therapeutic targets, in addition to information regarding new roles of amino acids that have yet to be discovered. As new functions are demonstrated, amino acid metabolism will become increasingly more important. For example, a study recently found that amino acids can modify protein targets on the lysine residue, namely aminoacylation, which transduces amino acid signals to regulate cellular functions (He et al., 2018). It should be expected that many new fields or breakthroughs will appear in the future.

## Author Contributions

CC, WY, and PY designed the content of the review with input from all the co-authors. ZW and XL wrote the original review draft. ZW and CC revised the article with feedback from all the co-authors. All authors approved this work for publication.

## Conflict of Interest

The authors declare that the research was conducted in the absence of any commercial or financial relationships that could be construed as a potential conflict of interest.
